# A trajectory study of postoperative frailty and cognitive function in elderly spinal surgery patients: a cohort study in China

**DOI:** 10.3389/fnagi.2025.1554901

**Published:** 2025-11-05

**Authors:** Rui Li, Qian Deng, Cheng Peng, Hong Song, Xiaotian Shan, Yao Wang, Chunyan Jin

**Affiliations:** ^1^Second Affiliated Hospital, Xuzhou Medical University, Xuzhou, China; ^2^Chongqing General Hospital, Chongqing, China

**Keywords:** elderly spinal surgery, debilitation, cognitive function, latent variable growth model, cross-lag model, nursing care

## Abstract

**Objective:**

To explore the developmental trajectory of postoperative frailty and cognitive function in elderly spinal surgery patients and the predictive relationship between the two.

**Methods:**

A total of 266 elderly individuals were selected as the study subjects, and their debilitation and cognitive functions were tracked and investigated at T1 (at the time of discharge from the hospital), T2 (at 3 months postoperatively), and T3 (at 6 months postoperatively), and the data were analyzed using cross-lagged model and latent variable growth model.

**Results:**

The latent variable growth model showed a decreasing trend in frailty (S = −0.197, *p* = 0.013) and an increasing trend in postoperative cognitive function (S = 0.124, *p* = 0.002) from T1 to T3 in elderly spinal surgery patients; at the initial level, frailty was negatively correlated with cognitive function (*r* = −0.452, *p* < 0.001), and initial levels of frailty negatively predicted the self development rate (*β* = −0.516, *p* < 0.001), the initial level of frailty negatively predicted the development rate of postoperative cognitive function (*β* = −0.321, *p* = 0.003), and the initial level of postoperative cognitive function positively predicted the development rate of frailty (*β* = 0.192, *p* = 0.031).

**Conclusion:**

Elderly spinal surgery patients showed a decreasing trend of postoperative frailty and an increasing trend of postoperative cognitive function, frailty and postoperative cognitive function at the initial level had a mutual predictive effect, the initial level of frailty was able to negatively predict the rate of development of their own and postoperative cognitive function, and the initial level of postoperative cognitive function was able to positively predict the rate of development of frailty.

## Introduction

With the advancement of medical conditions and the improvement of people’s living standards, life expectancy is increasing, leading to the gradual acceleration of the aging process of the population. The 21st century has already become the era of population aging, and China, as the world’s largest country in terms of population, ranks first in the world in terms of the size of the elderly population and its growth rate. The results of the seventh national census report suggest that the total number of people over 65 years old in China is as high as 190 million, accounting for 13.50%, up 4.63% year-on-year compared with the sixth census (Wu et al., 2021). A relevant study predicts that as of 2050, the proportion of the global population aged over 65 will be as high as 17% (North and Fiske, 2015), which means that social healthcare and security system will face great challenges in the coming decades. The functional deterioration of the skeletal system associated with aging has made the elderly a key population for herniated discs and vertebral fractures, and the number of elective spine surgeries for the elderly has increased year by year (Wang et al., 2015; Sanusi et al., 2024). In recent years, the concept of frailty is evolving in the field of geriatric research and has received extensive attention from national and international researchers. Frailty is a nonspecific state of increased vulnerability and reduced stress resistance in the elderly due to a decrease in physiological reserve capacity, which is characterized by individual dependence and an increased risk of adverse clinical health outcomes (Proietti and Cesari, 2020; Cesari et al., 2017), highly susceptible to adverse events such as falls, incapacitation, acute illness, hospitalization, medical problems, and death, placing an enormous burden on patients, families, and society (Wijnant et al., 2023; Bellelli et al., 2023). Postoperative cognitive dysfunction (POCD) is a mild impairment of higher cortical functions such as memory, concentration, and information processing that occurs after anesthesia and surgery, often occurring 1 week or later after surgery, and can last for weeks, months, or even years (Varpaei et al., 2024; Wu et al., 2023). POCD occurs in elderly patients undergoing orthopedic and other surgical procedures, and the incidence of POCD in surgical procedures has been reported to range from 25.8 to 53% 1 to 2 weeks after surgery, from 9.9 to 39% 1 to 3 months after surgery, and from 24% 6 months after surgery (Takazawa et al., 2023); in the study of elderly spinal surgery patients, the incidence of POCD at 1 week postoperatively in elderly patients over 65 was 23% (Kim et al., 2016). Cognitive impairment causes psychological distress and daily life inconvenience to elderly patients, and can lead to a decline in social activities and self-care ability. Therefore, postoperative cognitive dysfunction may aggravate patients’ physical frailty. In addition, it has been shown (Robinson et al., 2022) that physical frailty in turn acts on POCD and becomes one of the risk factors affecting POCD. A bidirectional and potentially synergistic relationship may exist between cognitive function and frailty in elderly patients undergoing spinal surgery: cognitive decline may accelerate frailty progression, while frailty status may in turn exacerbate cognitive dysfunction, creating a self-perpetuating cycle of decline. In summary, the varied findings of previous studies have led to an inability to accurately capture the predictive relationship between frailty and POCD and its direction. Therefore, further exploration of the dynamic relationship between the two during individual development is warranted. In contrast to linear regression models, which only reflect static relationships among multivariate variables, latent variable growth models are able to examine the developmental trajectories and dynamic interaction patterns among multivariate variables and pay attention to inter-individual differences. To summarize, the present study measured the frailty and POCD of elderly spinal surgery patients based on three time points, and established cross-lagged models and latent variable growth models to better explain the trend of the two variables and their interaction patterns from a dynamic perspective. The basic hypotheses are as follows: (1) the relationship between frailty and POCD in elderly spinal surgery patients at the three time points is longitudinally and mutually predictive; (2) the initial levels of the developmental trajectories of frailty and POCD in elderly spinal surgery patients can mutually predict each other’s developmental rates.

## Subjects and methods of study

### Research target

Two hundred and sixty-six elderly spinal surgery patients admitted to the Department of Orthopedics of multiple hospitals from January 2023 to December 2023 were selected as study subjects. Inclusion criteria: (1) patients met the clinical indications for spinal surgery, including compression fracture, intervertebral disc stenosis or herniation, etc.; (2) aged 60 years or older; (3) with basic understanding and communication ability, all signed the informed consent form. Exclusion criteria: (1) preoperative presence of mobility disorders or cognitive dysfunction; (2) previous history of malignant tumors, mental, psychological and other diseases. Elimination criteria: The patient died unexpectedly or withdrew for other reasons during the study. This study was conducted as a clinical research after approval by the ethics committee of the Second Affiliated Hospital of Xuzhou Medical University (approval number: [2022]1200026). The sample size calculation was based on the methodology described by Liu et al. (2014). When employing the Bayesian Information Criterion (BIC) as the primary model selection index, a minimum sample size of 200 is required. Accounting for an anticipated 20% attrition rate in longitudinal follow-up, the calculated sample size was *n* = 200/(1–0.2) = 250. In the current study, a total of 266 eligible patients were initially enrolled, and 232 participants successfully completed the trial protocol. This final sample size (*n* = 232) adequately meets the predetermined requirement (≥200) for statistical power.

## Research methodology

### Sample size calculation

According to the sample size requirement of the latent variable growth model, at least 200 patients were needed, and considering the possible high dropout rate of three measurements, the dropout rate was set at 15%, so the minimum sample size for this study was set at *n* = 200/(1–15%) = 235 cases.

### Survey instruments

1. The basic information was obtained through a self-made questionnaire, which included general demographic data such as age, gender, marital status, monthly income, educational background, occupation, BMI index, previous disease history, and nutritional status (NRS-2002).
2. Elderly inpatients’ frailty assessment scale (Niu et al., 2022) The scale was developed by scholar Niu Juanjuan and other scholars and completed the reliability test, including four dimensions: activities of living (6 items), general health status (5 items), nutritional status (3 items), and mental and psychological state (5 items), totaling 19 items. The item “Activities of Living” was scored on a 4-level scale of 0 to 1, while the remaining items were scored on a 2-level scale of 0 or 1, with a total score ranging from 0 to 19. The higher the score, the more severe the frailty condition of the patient is indicated. The Cronbach’s *α* coefficient of the total scale was 0.934, the test–retest reliability was 0.809, and the content validity was 0.964. In this study, the Cronbach’s α coefficients of this scale were 0.890, 0.862, and 0.834.
3. Montreal cognitive assessment scale (MoCA), which was revised by Nasreddine et al. (2005). The scale consists of eight cognitive domains, namely visuospatial structure (5 points), executive ability (3 points), attention (6 points), memory (3 points), language function (3 points), abstract thinking (2 points), calculation (5 points), and orienting (6 points), and the total score is the summation of scores in each cognitive domain, with scores ranging from 0 to 30 points, and if the years of schooling are ≤12 years, the higher the score, the better the cognitive functioning was suggested. The Cronbach’s *α* coefficient of the total scale was 0.85 and the split-half reliability was 0.80. In this study, the Cronbach’s α coefficients of this scale were 0.835, 0.840, and 0.822.

### Data recovery methods

The questionnaire for this study was administered face-to-face in the orthopedic ward after obtaining hospital and patient consent. The basic information questionnaire was obtained at the time of the patient’s first diagnostic admission, and according to previous studies (Moller et al., 1998; Miller et al., 2018; Yousuf et al., 2020), the prevalence rate of patients was about 25.8 to 53% at 1 to 2 weeks postoperatively, 9.9 to 39% at 1 to 3 months postoperatively, and 24% at 6 months postoperatively, and the debilitation assessment scale and cognitive assessment scale were obtained at the following times, respectively: T1 (at the time of discharge from the hospital), T2 (at 3 months postoperatively), and T3 (at 6 months postoperatively). To ensure patients privacy, the investigation was conducted in a confidential setting, and all patients signed an informed consent form. Patients with low literacy and dyslexia were asked to repeat the scale entries by the investigator, and the patients made independent choices. The consistency test of the questionnaire was conducted by setting polygraph questions, placing three questions with the same stem and different order of options in different positions of the questionnaire, and eliminating invalid questionnaires with different options in order to ensure the reliability of the questionnaire and to ensure that each questionnaire can express the true will of the patient. A total of 266 questionnaires were initially distributed, and 232 valid consecutive questionnaires were recovered at the completion of the three time points, with an effective recovery rate of 87.22%.

### Statistical methods

Correlation statistical analysis was performed using SPSS 26.0 and Mplus 8.0 software. The measurement data satisfying the normal distribution were expressed as mean ± standard deviation, and the count data were expressed as number of cases/percentage. Pearson correlation analysis was used for the correlation test. The missing data is supplemented by using the multiple interpolation method. Latent Growth Modeling (LGM) was used to explore the changes of debilitation and POCD in elderly spinal surgery patients, with the intercept indicating the initial level of debilitation or POCD, and the slope indicating the changes of debilitation or POCD, and to construct parallel latent growth models. A cross-lagged model was used to analyze the interaction of debilitation and POCD over time. A robust maximum likelihood Robust estimator (MLR) was used to evaluate the model. Fitting index: The chi-square to degrees of freedom ratio (*χ*^2^/df) is usually acceptable if it is less than 5, and a Goodness-of-Fit Index (CFI) >0.90 indicates a good fit. A Tucker–Lewis Index (TLI) >0.90 indicates a good fit, and a Root Mean Square Error of Approximation (RMSEA) < 0.08 is acceptable. It is better for the Standardized Root Mean Square Residual (SRMR) to be less than 0.08.

## Results

### General demographic information

A total of 232 valid questionnaires were collected in this study, Table 1.

**Table 1:** General information of respondents (*n* = 232).

Patient information	Classification	*N*	%	Patient information	Classification	*N*	%
Gender	Male	135	58.19	Lifestyle	Living alone	27	11.64
	Female	97	41.81		Family residence	205	88.36
Age (years)	60 ~ 69	45	19.40	BMI index	<18.5	47	20.26
	70 ~ 79	133	57.33		18.5 ~ 23.9	121	52.16
	≥80	54	23.27		≥24	64	27.58
Marriage	Unmarried	5	2.16	Hypertension	Yes	74	31.90
	Married	211	90.95		No	158	68.10
	Divorced/widowed	16	6.89	Coronary heart disease	Yes	57	24.57
Level of education	Primary school and below	85	36.64		No	175	75.43
	Junior high	89	38.36	Diabetes	Yes	54	23.28
	High school	45	19.40		No	178	76.72
	College and above	13	5.60	Osteoporosis	Yes	69	29.74
Place of residence	Town	123	53.02		No	117	50.43
	Rural	109	46.98		Unknown	46	19.83
Monthly household income (yuan)	<4,000	102	43.97	NRS-2002 (score)	<3	181	78.02
	4,000 ~ 6,000	84	36.21		≥3	51	21.98
	6,001 ~ 10,000	35	15.09				
	>10,000	11	4.73				

The gold standard for the diagnosis of osteoporosis is bone mineral density (BMD) measured by dual energy X-ray absorptiometry (DXA) with a *T*-score ≤−2.5.

### Frailty and POCD scores at three time points and correlation analysis in elderly spine surgery patients

Pearson correlation analyses were conducted to examine the bivariate relationships between cognitive function and frailty across all three assessment time points (Table 2). The results demonstrated statistically significant correlations between these variables at each time point (all *p* < 0.05), thereby satisfying the fundamental assumptions for subsequent cross-lagged panel modeling and parallel process latent growth curve analyses. The complete correlation matrix is presented in Table 2.

**Table 2:** Correlation analysis between frailty and POCD at three time points in elderly spinal surgery patients (*r*-value, *n* = 232).

Item	*M*	SD	1	2	3	4	5	6
1. Frailty T1	9.22	1.72	1					
2. Frailty T2	7.71	1.63	0.732^**^	1				
3. Frailty T3	7.37	1.54	0.633^**^	0.682^**^	1			
4. POCD T1	24.84	1.69	−0.321^**^	−0.369^**^	−0.399^**^	1		
5. POCD T2	25.22	1.76	−0.417^**^	−0.422^**^	−0.421^**^	0.733^**^	1	
6. POCD T3	26.56	1.22	−0.343^**^	−0.416^**^	−0.402^**^	0.502^**^	0.589^**^	1

^**^Significant correlation at the 0.01 level (two-tailed).

### Cross-lagged modeling of frailty and POCD in elderly spinal surgery patients

**Figure 1:**
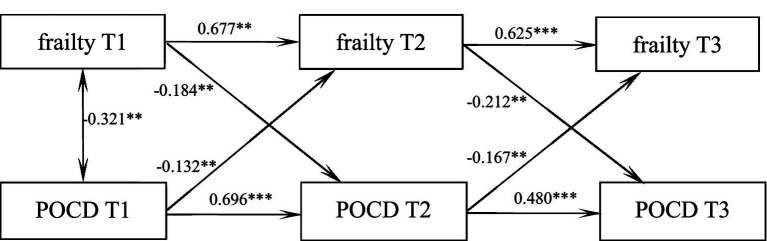
Pathways of predicted role of frailty and POCD in elderly spinal surgery patients at three time points. ***p < 0.001, **p < 0.01.

Four path models (M1–M4) were constructed to examine the causal relationship between cognitive function and frailty: (1) M1 (baseline model): an autoregressive path containing only cognitive function and frailty (T1 → T2 → T3). M2 (One-way Prediction Model): on the basis of M1, add the prediction path of cognitive function for frailty at the next time point (T1_ cognitive function → T2_ frailty, T2_ cognitive function → T3_ frailty); M3 (Reverse Prediction Model): On the basis of M1, add the prediction paths of frailty for cognitive function at the next time point (T1_ frailty → T2_ cognitive function, T2_ frailty → T3_ cognitive function); M4 (Bidirectional Full Model): it simultaneously includes the bidirectional paths of cognitive function → frailty and frailty → cognitive function. Robust maximum likelihood estimation (MLR) was adopted to compare the fitting indicators. M4 was significantly superior to M1, M2, and M3 (Δχ^2^ = 122.362, *p* < 0.001), and had the best fitting (CFI > 0.98, RMSEA<0.05), supporting the hypothesis of their interaction. A cross-lagged model was developed to examine the reciprocal predictive relationship between frailty and POCD. The model fit well, *χ*^2^/df = 2.070, GFI = 0.972, TLI = 0.922, and RMSEA = 0.128. As shown in Figure 1: frailty and POCD mutually predicted each other in the initial level condition(*r* = −0.321, *p* = 0.002); Moreover, the level of frailty, on average, significantly and positively predicted POCD in the next node(T1 → T2: *β* = −0.184, *p* = 0.005; T2 → T3: *β* = −0.212, *p* = 0.003), and the level of POCD likewise significantly and positively predicted the level of frailty in the next node(T1 → T2: *β* = −0.132, *p* = 0.008; T2 → T3: *β* = −0.167, *p* = 0.006), Figure 1.

### Parallel latent variables for debilitation and POCD in elderly spine surgery patients

In this study, a two-process parallel latent variable growth model was constructed to model the development trajectories of cognitive function and frailty respectively: the cognitive function model showed a linear upward trend (based on the three measurement time points T1, T2, and T3, with an interval of 3 months). The frailty model shows a linear downward trend (based on the three measurement time points T1, T2, and T3, with an interval of 3 months). The specific factor loading Settings are shown in Table 3.

**Table 3:** Factor loading settings.

Latent variables	Measuring time point	Load setting	Theoretical basis
Cognitive function intercept (I cognitive function)	T1/T2/T3	1/1/1	Reflect baseline level of cognitive function
Cognitive function slope (S cognitive function)	T1/T2/T3	0/1/2	Linear rate of change
Frailty intercept (I frailty)	T1/T2/T3	1/1/1	Reflect baseline level of frailty
Frailty slope (S frailty)	T1/T2/T3	0/1/2	Linear rate of change

### Developmental trajectory of debilitation in elderly spinal surgery patients

**Figure 2:**
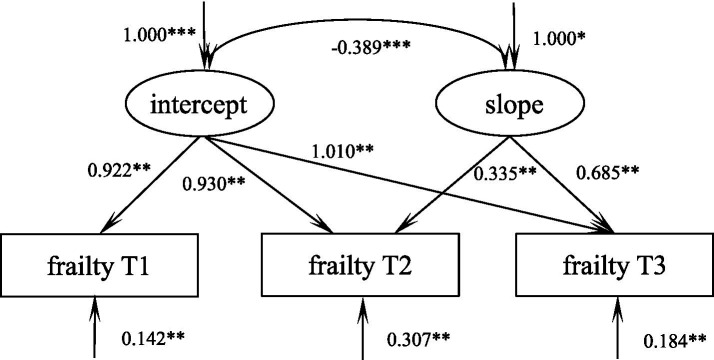
Model of debilitation in elderly spinal surgery patients. ***p < 0.001, **p < 0.01, *p < 0.05.

According to the unconditional latent variable linear growth model of debilitation in elderly spinal surgery patients, the fit indices were as follows: *χ*^2^/df = 0.084, GFI = 1.000, TLI = 1.006, RMSEA = 0.000, SRMR = 0.003, which is a good fit. The model intercept, i.e., the initial value of frailty of 9.12, showed a decreasing trend in the subsequent three measurements (S = −0.197, *p* = 0.013), and there was a significant correlation between the intercept and the slope (*r* = −0.389, *p* < 0.001), suggesting that there is a significant negative correlation between the initial state of frailty in elderly spinal surgery patients and the rate of development, i.e., the higher the initial level of frailty in elderly spinal surgery patients, the slower the rate of decline in the later stages, Figure 2.

### Trajectory of POCD in elderly spine surgery patients

**Figure 3:**
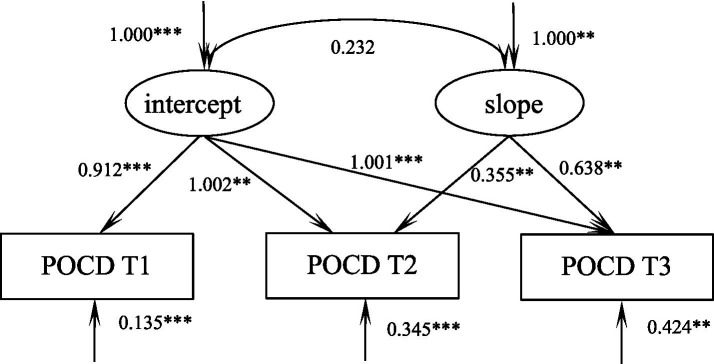
Model of POCD in elderly spinal surgery patients. ***p < 0.001, **p < 0.01.

According to the unconditional latent variable linear growth model of POCD in elderly spinal surgery patients, the fit indices were as follows: *χ*^2^/df = 7.786, GFI = 0.978, TLI = 0.935, RMSEA = 0.066, and SRMR = 0.032, which is a good fit. The model intercept, i.e., the initial value of POCD of 24.50, showed an increasing trend in the subsequent three measurements (S = 0.124, *p* = 0.002), and the correlation between intercept and slope was not significant (*r* = 0.232, *p* = 0.175), suggesting that there is no significant positive correlation between the initial state of POCD in elderly patients undergoing spinal surgery and the speed of its development, as shown in Figure 3.

### Dynamic relationship between frailty and POCD in elderly spinal surgery patients

**Figure 4:**
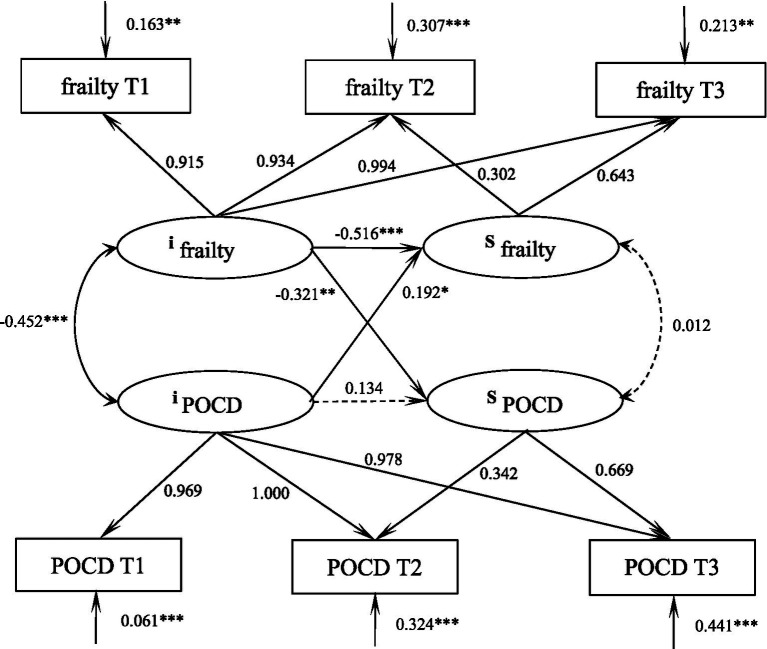
Parallel latent variable modeling of frailty and POCD in elderly spinal surgery patients. ***p < 0.001, **p < 0.01.

The parallel latent variable growth model of debilitation and POCD in elderly spinal surgery patients was constructed, and the fitting indexes were as follows: *χ*^2^/df = 2.048, GFI = 0.990, TLI = 0.979, RMSEA = 0.065, and SRMR = 0.036, which was a good fit. At the initial level, frailty was negatively correlated with POCD (*r* = −0.452, *p* < 0.001), i.e., the higher the patient’s level of frailty, the lower his or her level of POCD. The initial level of frailty negatively predicted its own development rate (*β* = −0.516, *p* < 0.001), i.e., the higher the initial level of frailty, the slower the rate of decline; the initial level of frailty was able to negatively predict the rate of development of POCD (*β* = −0.321, *p* = 0.003), i.e., the higher the initial level of frailty, the slower the rise of the level of POCD in the patient, and the postoperative cognitive The initial level of functioning positively predicted the rate of development of frailty (*β* = 0.192, *p* = 0.031), i.e., the higher the initial level of POCD, the faster the rate of decline of frailty, and the path of action is shown in Figure 4.

## Discussion

### Interrelationship between frailty and POCD in elderly spinal surgery patients

In this study, by constructing a cross-lagged model, frailty and postoperative cognitive dysfunction (POCD) were found to be mutually predictive under the initial level condition; moreover, the level of frailty on average significantly and positively predicted POCD at the next node, and the level of POCD likewise significantly and positively predicted the level of frailty at the next node. Previous studies have shown that the incidence of frailty in elderly hospitalized patients is as high as 39.3% (Veronese et al., 2021) with a 20.35% incidence of cognitive decline (Yuan et al., 2021). Previous studies have described the cross-sectional relationship between frailty and POCD (Ma et al., 2022) which confirmed a significant correlation between frailty and POCD, but could not confirm a causal link. Nari et al. (2021) found that frail older adults are more likely to experience cognitive impairment. This study revealed a bidirectional predictive relationship between frailty and cognitive function in elderly patients undergoing spinal surgery. Higher baseline frailty levels significantly predicted subsequent cognitive decline, potentially mediated through multiple pathways: (1) frail individuals experience reduced physical activity and social engagement due to functional limitations, compounded by surgical/anesthetic effects that further impair cognition; (2) frailty-associated comorbidities (e.g., micronutrient deficiencies, vascular dysfunction) may synergistically compromise cerebral perfusion and neurotransmitter homeostasis. Conversely, postoperative cognitive function also predicted subsequent frailty progression, suggesting a vicious cycle. These findings align with Yuan et al.’s (2023) research, demonstrating that early-stage frailty contributes to cognitive impairment, which in turn exacerbates physical frailty progression. This suggests that it is important for clinical staff to emphasize the assessment of frailty and POCD levels in elderly spinal surgery patients, to take advantage of the interactive relationship between the two, and to actively intervene in controllable factors in order to delay frailty and correct cognitive impairment.

### Trajectories and dynamic relationships between frailty and POCD in elderly spinal surgery patients

In this study, a latent variable growth model revealed that elderly spinal surgery patients showed a decreasing trend in the level of postoperative frailty and an increasing trend in both levels of cognitive function. Previous studies have disputed the trend of frailty in postoperative patients, and a longitudinal investigation showed that (Chen et al., 2022) postoperative patient frailty showed a decreasing trend, similar to the results of this study. However, Chen et al.’s (2022) study showed a nonsignificant trend in postoperative debilitation. Frailty trends varied depending on the type of disease, surgical procedure, demographics, and measurement tools used in the sample population. The reason for the gradual decrease in the level of postoperative frailty in elderly spinal surgery patients may be that, on the one hand, the trauma of the disease and the surgery may have limited the patient’s ability to care for themselves, which resulted in relatively significant frailty, and on the other hand, as the patient recovered from the surgery, the patient’s ability to care for themselves and their mobility increased, and the level of frailty was suppressed. However, the trend of previous studies regarding POCD was relatively consistent (Glumac et al., 2021; Kannampallil et al., 2021) and as in the present study, there was an upward trend in POCD. Cognitive function in elderly spinal surgery patients is affected by many factors such as anesthesia and surgery, and postoperative cognitive dysfunction may occur, which improves with the recovery of body metabolism and physical function.

The results of the parallel latent variable growth model showed that frailty was negatively correlated with cognitive function at the initial level, i.e., the higher the patient’s level of frailty, the lower their level of POCD. This has been confirmed several times in previous studies (Panza et al., 2018). The initial level of frailty negatively predicts its own rate of progression, i.e., the higher the initial level of frailty, the slower the rate of decline, for the understandable reason that the more debilitated the patient is, the longer the recovery time and the slower the rate of recovery after trauma. This was also confirmed by McIsaac et al. (2020). The initial level of frailty negatively predicts the rate of development of POCD, i.e., the higher the initial level of frailty, the slower the level of POCD rises.

Clinical practitioners should prioritize comprehensive frailty assessment in elderly patients scheduled for spinal surgery, with particular attention to those identified as high-risk through standardized screening tools such as the Fried frailty phenotype. For vulnerable patients, implementation of a structured, phased rehabilitation protocol beginning in the immediate postoperative period is recommended. This intervention should progress systematically from initial bed-based joint mobilization and respiratory exercises to more advanced resistance training and balance activities, complemented by targeted nutritional support including protein optimization and vitamin D supplementation.

Patients exhibiting postoperative cognitive impairment may benefit from integrated cognitive rehabilitation strategies combining computerized cognitive training with reality orientation therapy. To ensure optimal outcomes, implementation of a rigorous monthly monitoring system utilizing both frailty indices and MoCA cognitive assessments is advised. This approach enables timely intensification of exercise regimens upon detecting cognitive improvement, thereby creating a dynamic, bidirectional intervention framework that simultaneously addresses both cognitive and physical frailty components. Current evidence suggests this comprehensive approach can significantly enhance functional recovery outcomes in this high-risk population.

### Shortcomings and prospects

This study was designed longitudinally and investigated the changing trajectories of frailty and cognitive function within 6 months after surgery in 232 elderly patients undergoing spinal surgery, which can provide a basis for dynamically understanding the changes in clinical practice. Nevertheless, this study has several limitations that warrant consideration. First, the inherent challenge in detecting early-stage frailty—a clinically subtle condition—was compounded by the absence of standardized diagnostic criteria, potentially increasing measurement variability. Additionally, the longitudinal design was constrained by relatively brief follow-up intervals and a single-center setting with limited sample size, factors that may have attenuated statistical power. Future investigations should employ more sensitive assessment instruments, extend observation periods, incorporate multicenter recruitment strategies, and optimize methodological rigor to better elucidate the bidirectional relationship between cognitive function and frailty progression.

## Conclusion

The study revealed distinct trajectories in postoperative recovery among elderly spinal surgery patients: frailty scores demonstrated a significant decreasing trend during the 6-month postoperative period, while cognitive function exhibited progressive improvement. Importantly, baseline assessments showed bidirectional predictive relationships between these outcomes. Higher initial frailty levels were associated with slower recovery trajectories for both frailty reduction and cognitive improvement. Conversely, better baseline cognitive function predicted more rapid frailty resolution. These findings highlight the dynamic interplay between physical and cognitive recovery processes. Future research directions include implementing more sensitive assessment protocols, extending longitudinal observation periods, and conducting multicenter studies to further elucidate the complex relationship between cognitive function and frailty progression in this vulnerable population.

## Data Availability

The original contributions presented in the study are included in the article/supplementary material, further inquiries can be directed to the corresponding author.

## References

[ref1] Bellelli F. Consorti E. Hettiarachchige T. Hettiarachchige T. M. K. Rossi P. Lucchi T.. (2023). Relationship among age, education and frailty in older persons. J. Frailty Aging 12, 326–328. doi: 10.14283/jfa.2023.39, PMID: 38008985

[ref2] Cesari M. Calvani R. Marzetti E. (2017). Frailty in older persons. Clin. Geriatr. Med. 33, 293–303. doi: 10.1016/j.cger.2017.02.002, PMID: 28689563

[ref3] Chen W. Y. Liu C. Y. Shih C. C. Chen Y.-S. Cheng H.-W. Chiou A.-F. (2022). Factors associated with frailty in patients undergoing cardiac surgery: a longitudinal study. J. Cardiovasc. Nurs. 37, 204–212. doi: 10.1097/JCN.0000000000000787, PMID: 34145204

[ref4] Glumac S. Kardum G. Sodic L. Bulat C. Covic I. Carev M.. (2021). Longitudinal assessment of preoperative dexamethasone administration on cognitive function after cardiac surgery: a 4-year follow-up of a randomized controlled trial. BMC Anesthesiol. 21:129. doi: 10.1186/s12871-021-01348-z, PMID: 33892653 PMC8063389

[ref5] Kannampallil T. Holzer K. J. Abraham J. Naim U. Lenze E. J. Haroutounian S.. (2021). Surgical complications in older adults predict decline in self-perceived cognitive function in the ensuing year: a cohort study. Am. J. Geriatr. Psychiatry 29, 352–361. doi: 10.1016/j.jagp.2020.09.007, PMID: 32981851

[ref6] Kim J. Shim J. K. Song J. W. Kim E. K. Kwak Y. L. (2016). Postoperative cognitive dysfunction and the change of regional cerebral oxygen saturation in elderly patients undergoing spinal surgery. Anesth. Analg. 123, 436–444. doi: 10.1213/ANE.0000000000001352, PMID: 27285000

[ref7] Liu Y. Luo F. Liu H. (2014). Influencing factors of the multi-stage mixed growth model: distance and morphology. Acta Psychol. Sin. 46, 1400–1412. doi: 10.3724/SP.J.1041.2014.01400

[ref8] Ma W. Wu B. Gao X. Zhong R. (2022). Association between frailty and cognitive function in older Chinese people: a moderated mediation of social relationships and depressive symptoms. J. Affect. Disord. 316, 223–232. doi: 10.1016/j.jad.2022.08.032, PMID: 35988782

[ref9] McIsaac D. Taljaard M. Bryson G. McIsaac D. I. Bryson G. L. Beaulé P. E.. (2020). Frailty as a predictor of death or new disability after surgery: a prospective cohort study. Ann. Surg. 271, 283–289. doi: 10.1097/SLA.0000000000002967, PMID: 30048320

[ref10] Miller D. Lewis S. R. Pritchard M. W. Schofield-Robinson O. J. Shelton C. L. Alderson P.. (2018). Intravenous versus inhalational maintenance of anaesthesia for postoperative cognitive outcomes in elderly people undergoing non-cardiac surgery. Cochrane Database Syst. Rev. 8:CD12317. doi: 10.1002/14651858.CD012317.pub2 PMC6513211 30129968

[ref11] Moller J. T. Cluitmans P. Rasmussen L. S. Houx P. Rasmussen H. Canet J.. (1998). Long-term postoperative cognitive dysfunction in the elderly ISPOCD1 study. ISPOCD investigators. International study of post-operative cognitive dysfunction. Lancet 351, 857–861. doi: 10.1016/S0140-6736(97)07382-0, PMID: 9525362

[ref12] Nari F. Jang B. N. Youn H. M. Jeong W. Jang S. I. Park E. C. (2021). Frailty transitions and cognitive function among south Korean older adults. Sci. Rep. 11:10658. doi: 10.1038/s41598-021-90125-6, PMID: 34017031 PMC8138002

[ref13] Nasreddine Z. S. Phillips N. A. Bedirian V. Charbonneau S. Whitehead V. Collin I.. (2005). The Montreal cognitive assessment, MoCA: a brief screening tool for mild cognitive impairment. J. Am. Geriatr. Soc. 53, 695–699. doi: 10.1111/j.1532-5415.2005.53221.x, PMID: 15817019

[ref14] Niu J. Shi M. Zhang S. Zhang J. (2022). Development and reliability and validity test of the frailty assessment scale for elderly inpatients. Nurs. Res. 36, 402–407. doi: 10.12102/j.issn.1009-6493.2022.03.004

[ref15] North M. S. Fiske S. T. (2015). Modern attitudes toward older adults in the aging world: a cross-cultural meta-analysis. Psychol. Bull. 141, 993–1021. doi: 10.1037/a0039469, PMID: 26191955

[ref16] Panza F. Lozupone M. Solfrizzi V. Sardone R. Dibello V. di Lena L.. (2018). Different cognitive frailty models and health- and cognitive-related outcomes in older age: from epidemiology to prevention. J Alzheimer's Dis 62, 993–1012. doi: 10.3233/JAD-170963, PMID: 29562543 PMC5870024

[ref17] Proietti M. Cesari M. (2020). Frailty: what is it? Adv. Exp. Med. Biol. 1216, 1–7. doi: 10.1007/978-3-030-33330-0_1, PMID: 31894541

[ref18] Robinson T. L. Gogniat M. A. Miller L. S. (2022). Frailty and cognitive function in older adults: a systematic review and Meta-analysis of cross-sectional studies. Neuropsychol. Rev. 32, 274–293. doi: 10.1007/s11065-021-09497-1, PMID: 33886024

[ref19] Sanusi T. D. Momin S. Sachdev B. Leung A. (2024). Super-elderly, spinal surgery, evaluating the risks and benefits: a retrospective single-Centre cohort study. Acta Neurochir. 166:248. doi: 10.1007/s00701-024-06135-6, PMID: 38833175

[ref20] Takazawa T. Horiuchi T. Orihara M. Nagumo K. Tomioka A. Ideno Y.. (2023). Prevention of postoperative cognitive dysfunction by minocycline in elderly patients after total knee arthroplasty: a randomized, double-blind, placebo-controlled clinical trial. Anesthesiology 138, 172–183. doi: 10.1097/ALN.0000000000004439, PMID: 36538374

[ref21] Varpaei H. A. Farhadi K. Mohammadi M. Khafaee pour khamseh A. Mokhtari T. (2024). Postoperative cognitive dysfunction: a concept analysis. Aging Clin. Exp. Res. 36:133. doi: 10.1007/s40520-024-02779-7, PMID: 38902462 PMC11189971

[ref22] Veronese N. Custodero C. Cella A. Demurtas J. Zora S. Maggi S.. (2021). Prevalence of multidimensional frailty and pre-frailty in older people in different settings: a systematic review and meta-analysis. Ageing Res. Rev. 72:101498. doi: 10.1016/j.arr.2021.101498, PMID: 34700009 PMC12149324

[ref23] Wang M. Y. Widi G. Levi A. D. (2015). The safety profile of lumbar spinal surgery in elderly patients 85 years and older. Neurosurg. Focus. 39:E3. doi: 10.3171/2015.7.FOCUS15180, PMID: 26424343

[ref24] Wijnant S. Benz E. Luik A. I. Wijnant S. R. A. Rivadeneira F. Voortman T.. (2023). Frailty transitions in older persons with lung function impairment: a population-based study. J. Gerontol. A Biol. Sci. Med. Sci. 78, 349–356. doi: 10.1093/gerona/glac202, PMID: 36226677 PMC9951055

[ref25] Wu L. Huang Z. Pan Z. (2021). The spatiality and driving forces of population ageing in China. PLoS One 16:e243559. doi: 10.1371/journal.pone.0243559, PMID: 33428682 PMC7799793

[ref26] Wu W. F. Lin J. T. Qiu Y. K. Dong W. Wan J. Li S.. (2023). The role of epigenetic modification in postoperative cognitive dysfunction. Ageing Res. Rev. 89:101983. doi: 10.1016/j.arr.2023.101983, PMID: 37321381

[ref27] Yousuf M. S. Samad K. Ullah H. (2020). Postoperative cognitive dysfunction following general Anaesthesia in patients undergoing elective non-cardiac surgery. J. Coll. Physicians Surg. Pak. 30, 417–419. doi: 10.29271/jcpsp.2020.04.417, PMID: 32513364

[ref28] Yuan L. Zhang X. Guo N. Li Z. Lv D. Wang H.. (2021). Prevalence of cognitive impairment in Chinese older inpatients and its relationship with 1-year adverse health outcomes: a multi-center cohort study. BMC Geriatr. 21:595. doi: 10.1186/s12877-021-02556-5, PMID: 34696723 PMC8543818

[ref29] Yuan Y. Peng C. Burr J. A. Lapane K. L. (2023). Frailty, cognitive impairment, and depressive symptoms in Chinese older adults: an eight-year multi-trajectory analysis. BMC Geriatr. 23:843. doi: 10.1186/s12877-023-04554-1, PMID: 38087208 PMC10717397

